# Inhibitory Effect of Monoterpenoid Glycosides Extracts from Peony Seed Meal on *Streptococcus suis* LuxS/AI-2 Quorum Sensing System and Biofilm

**DOI:** 10.3390/ijerph192316024

**Published:** 2022-11-30

**Authors:** Jinpeng Li, Yamin Shen, Jing Zuo, Shuji Gao, Haikun Wang, Yuxin Wang, Li Yi, Xiaogai Hou, Yang Wang

**Affiliations:** 1 College of Animal Science and Technology, Henan University of Science and Technology, Luoyang 471000, China; 2 Key Laboratory of Molecular Pathogen and Immunology of Animal of Luoyang, Luoyang 471000, China; 3 Animal Disease Prevention and Food Safety Key Laboratory of Sichuan Province, College of Life Sciences, Sichuan University, Chengdu 610064, China; 4 College of Life Science, Luoyang Normal University, Luoyang 471000, China; 5 College of Agriculture/College of Tree Peony, Henan University of Science and Technology, Luoyang 471000, China

**Keywords:** peony seed cake extract, paeoniflorin, *Streptococcus suis*, LuxS/AI-2 system, biofilm

## Abstract

*Streptococcus suis* LuxS/AI-2 quorum sensing system regulates biofilm formation, resulting in increased pathogenicity and drug resistance, and diminished efficacy of antibiotic treatment. The remaining peony seed cake after oil extraction is rich in monoterpenoid glycosides, which can inhibit the formation of bacterial biofilm. In this study, we investigated the effect of seven major monocomponents (suffruticosol A, suffruticosol B, suffruticosol C, paeonifloin, albiflorin, trans-ε-viniferin, gnetin H) of peony seed meal on minimum inhibitory concentration (MIC) and minimum bactericidal concentration (MBC) of *S. suis*. The results showed that the MICs of the seven single components were all greater than 200 μg/mL, with no significant bacteriostatic and bactericidal advantages. Crystal violet staining and scanning electron microscope observation showed that the seven single components had a certain inhibitory effect on the biofilm formation ability of *S. suis* at sub-MIC concentration. Among them, the ability of paeoniflorin to inhibit biofilm was significantly higher than that of the other six single components. AI-2 signaling molecules were detected by bioreporter strain *Vibrio harvey BB170*. The detection results of AI-2 signal molecules found that at 1/2 MIC concentration, paeoniflorin significantly inhibited the production of *S. suis* AI-2 signal, and the inhibitory effect was better than that of the other six single components. In addition, molecular docking analysis revealed that paeoniflorin had a significant binding activity with LuxS protein compared with the other six single components. The present study provides evidence that paeoniflorin plays a key role in the regulation of the inhibition of *S. suis* LuxS/AI-2 system and biofilm formation in peony seed meal.

## 1. Introduction

*Streptococcus suis* disease is a zoonotic disease caused by multiple pathogenic *S. suis* infections characterized by meningitis, arthritis, endocarditis, pneumonia, sepsis, and toxic shock syndrome [1]. *S. suis* mainly infects nursery pigs and weaned piglets, and then spreads from animal to animal or to human beings [2], which not only causes significant economic losses to the pig industry around the country but also poses a major threat to human life after causing meningitis and sepsis [3]. *S. suis* type 2 is a major pathogenic serotype of *S. suis* disease, which is typically pathogenic through colonization of the respiratory tract, penetration of the host mucosal barrier, transmission through blood, and invasion of different organs [4]. In addition, *S. suis* type 2 can secrete extracellular polymers such as polysaccharides, proteins, nucleic acids, lipids, and bacterial vesicles to promote the formation of biofilms and increase the resistance and immune evasion ability of bacteria when encountering adverse environments, such as stress and antibiotics [5].

When biofilms are formed, they are 10–1000 times more resistant to antibiotics than planktonic cells [6]. This may be due to a decrease in antibiotic permeability in the biofilm. The LuxS/AI-2 quorum sensing system can regulate *S. suis* colony morphology, bacterial virulence, antibiotic resistance, and biofilm formation, etc. The transcriptional expression levels of *S. suis* and Δ*luxS* strains were compared, and 29 genes were considered to be target genes regulated by LuxS/AI-2 quorum sensing system, which are related to bacterial virulence and iron uptake. The LuxS/AI-2 system regulates the metabolism and adhesion-related genes of *S. suis* by secreting AI-2 signaling molecules, enabling bacteria to aggregate with each other and influencing the secretion of polysaccharides, proteins, and other extracellular polymers (EPS), thus promoting biofilm formation [7]. The QS system mediated by LuxS/AI-2 quorum sensing is widely detected in Gram-positive and Gram-negative bacteria, and its signaling molecule AI-2 is considered to be an interspecific communication molecule [8]. AI-2 is a group of intertransforming self-inducible compounds derived from 4, 5-dihydroxy-2, 3-glutarone (DPD), which is synthesized by LuxS enzyme after spontaneous cycling to various furanones [9]. LuxS enzyme activity is influenced by its phosphorylation state, which is mediated by the serine/threonine kinase Stk1. Specific bacterial species can detect different forms of DPD as their active AI-2 signal. With different DPDS rapidly switching between each other, AI-2 provides a means of communication between species [8].

In *S. suis*, The LuxS protease in the methyl cycle regulates the synthesis of AI-2, which can be transported out of the cell. As *S. suis* divide and proliferate, the concentration of AI-2 increases. When AI-2 levels reach a threshold, bacteria “count” their surroundings by sensing the molecular density of AI-2. Thus, QS gene expression is regulated, including BF formation, drug resistance, and virulence-related gene expression [5]. Inhibition of biofilms through the use of medicinal plants seems to be an important approach and is considered a promising antibacterial or anti-biofilm agent [10].

Peony seed meal has a wide range of pharmacological effects, such as anti-inflammatory, anti-infection, anti-tumor, immune regulation, and inhibition of the quorum sensing system [11]. However, no clear components of the LuxS/AI-2 QS system of *S. suis* regulated by peony seed meal have been found. In this study, seven main components of peony seed meal (suffruticosol A, suffruticosol B, suffruticosol C, paeonifloin, albiflorin, trans-ε-viniferin, gnetin H) were used to detect AI-2 signaling molecules and BF inhibition test. To determine the specific single component of peony seed meal that inhibits the LuxS/AI-2 QS system and BF of *S. suis*, and to provide a new idea for further exploring the inhibition of *S. suis* by peony seed meal.

## 2. Materials and Methods

### 2.1. Bacterial Strains, Growth Conditions, and Reagents

*S. suis* HA9801, *V. harveyi* BB170 were used to study the anti-Luxs/AI-2 system and anti-biofilm mechanism of each single component of peony seed cake extract. *S. suis* type 2 strain was isolated from a sick pig in Hai‘an city, Jiangsu province. *S. suis* was grown aerobically at 37 °C in Todd-Hewitt broth (THB) or plated on THB agar supplemented with 5 % (vol/vol) sheep blood. *V. harveyi BB170* is kindly provided by Professor Xiangan Han and was grown in autoinducible bioassay medium (M2289, Topu Biological Engineering Co., Ltd., Shenzhen, China) at 28 °C. Suffruticosol A, Suffruticosol B, Suffruticosol C, albiflorin, paeonifloin, trans-ε-viniferin, gnetin H were kindly provided by Professor Pu Liu from Henan University of Science and Technology (Luo Yang, China).

### 2.2. Determination of MIC and MBC

Determination of MIC and MBC was carried out according to the two-fold serial dilution method [12], with minor modifications. Specifically, add 100 µL of THB to each well of a 96-well microtiter plate. A series of diluents were prepared from 1 to 12 to reduce the concentration to 3200 μg/mL–6.25 μg/mL by adding 100 µL of each plant extract to the first column of microtiter plates. Subsequently, 100 µL of the bacterial culture adjusted to 1 × 10^5^ CFU was transferred into each well. Minimum inhibitory concentration (MIC), the lowest concentration of each peony seed cake extract that completely inhibited the growth of *S. suis* in a 96 wells plate was the minimum inhibitory concentration. MBC was the lowest concentration of antibiotic at which there was no colony-forming unit on the THA.

### 2.3. Crystal Violet Staining Method to Detect Biofilm

The 96-well flat-bottomed polystyrene microtiter plate was used to evaluate the ability of plant extracts to inhibit the biofilm formation of *S. suis*, using the modified crystal violet quantitative method [13]. The overnight cultured strains were diluted to 1 × 10^5^ CFU/mL in THB medium, and 100 μL of bacterial suspension and 100 μL of sub-MIC plant extracts (1/32 × MIC–1/2 × MIC) were added into 96-well plates. After incubation at 37 °C for 24 h, the biofilm was detected by crystal violet staining. The blank control group did not add any plant extract.

Crystal violet (CV) staining procedure: The culture in the well was removed and washed three times with PBS to remove the planktonic culture, and then the remaining biofilm was dried at 50 °C for 30 min. Then, 200 μL of 0.1% crystal violet solution was added to each well for staining and incubated at room temperature for 15 min. Then, the crystal violet was removed from the well and rinsed three times with PBS. The microplate reader was zeroed out using a blank well before OD readings were taken. Absorbance was measured at 595 nm using a Multiskan SkyHigh full-wavelength microplate reader (A51119500C, ThermoFisher Scientific, Waltham, MA, USA), and percentage inhibition was quantified using Equation (1).

Biofilm inhibition rate (%) = (OD595 _blank control_ − OD595 _experimental group_)/OD595 _blank control_ (1)

### 2.4. Scanning Electron Microscopy of Biofilms

The biofilms of every single component of peony seed meal supplemented with 1/2MIC concentration of *S. suis* were observed. The cultures were diluted to reach a concentration of 10^6^ cfu/mL. Then, the *S. suis* of 2 mL was added to a sterile 12 wells plate (I0905, Corning, China) containing a cell climbing piece (0.4 cm^2^). After incubating at 37 °C for 24 h, the cell crawl sheet was gently washed with PBS (0.1 M, pH = 6.8) to remove planktonic bacteria. After being treated with 2.5% (*w/v*) glutaraldehyde for 2 h, the *S. suis* biofilm was lightly washed with sterile PBS (0.1M, pH = 6.8) and dehydrated in a gradient ethanol system (20, 30, 50, 60, 70, 80, 90, and 100% ethanol). The biofilm was metalized with a sputter coater (8 mA, 4 min) and observed by SEM (JSM-6380LV, Jeol, Tokyo, Japan).

### 2.5. AI-2 Activity Assay

AI-2 is a diester of furanyl borate. The synthesis of AI-2 in bacteria mainly involves two enzymatic steps. First, methioadenosine ribosidase, encoded by the pfs gene, removes the adenine of S-adenosine homocysteine (SAH) to produce S-ribose homocysteine (SRH). Then, SRH enzyme encoded by *luxS* gene converts SRH to homocysteine and 4,5-dihydroxy-2, 3-pentadione (DPD); Finally, the DPD rearranges spontaneously to form AI-2. One of *Vibrio harvey* quorum sensing systems (AI-2 signaling system) is a species non-specific system, and the sensor responds to AI-2 signaling molecules in a metroiluminescence manner. To determine the effect of suffruticosol A, suffruticosol B, suffruticosol C, paeonifloin, albiflorin, trans-ε-viniferin, gnetin H on the activity of AI-2 [14], *S. suis* HA9801 was grown overnight at 37 °C, the bacterial cultures were diluted to 10^5^ CFU/mL, and a single component of peony seed cake was added to each. Incubate at 37 °C for 12 h and centrifuge at 4 °C for 15 min at 12,000 g. The products were filtered by a 0.22 μm stream filter and stored at −80 °C for a long time.

In order to test the inhibitory ability of each peony seed cake extract on *S. suis* AI-2 activity, *V. harveyi* BB170 was cultured at 28 °C for 12 h, and the OD_600_ was adjusted to 0.8, and then diluted 5000 times. A total of 180 μL diluent and 20 μL AI-2 supernatant were incubated at 28 °C for 4 h in the dark, and bioluminescence values were measured with a bioluminescence photometer at 490 nm wavelength. The test results are shown in the form of ratio: luminescence value of each experimental group/luminescence value of *E. coli* DH5α.

### 2.6. Real-Time RT-PCR

RNA was obtained from cultures of each peony seed meal single component with *S. suis* using the RNA-easy Isolation Reagent (R701-02, Vazyme, Shanghai, China) following the kit instructions. *S. suis* was cultured with the extract of each peony seed meal supplemented with 1/2MIC for 12 h at 37 °C. Subsequently, the RNA samples of each group were converted into cDNA using HiScript Reverse Transcriptase (R101-01, Vazyme, China). The cDNA samples of each group were amplified by the ChamQ SYBR qPCR Master Mix (High ROX Premixed) (Q341-02, Vazyme, China). The experiment was carried out according to the reference instruction. The reference gene is 16S rRNA (Supplementary Table S1).

### 2.7. Molecular Docking Assay

Virtual molecular docking analysis was performed according to the previous research method [15] and slightly modified to check whether suffruticosol A, suffruticosol B, suffruticosol C, paeonifloin, albiflorin, trans-ε-viniferin, gnetin H interact with LuxS protein. LuxS proteins were crystallized and structurally resolved from previous studies in our laboratory [16]. The peony seed meal extract file was downloaded from PubChem (https://pubchem.ncbi.nlm.nih.gov/ (accessed on 12 April 2021)). SYBYL-X 2.1 software was used to analyze the maximum binding capacity between each peony seed cake extract and LuxS protein. The optimized small molecules of each peony seed cake extract were docked with LuxS protein model by Surflex-Dock program, and the score was high (Total score > 6) and is considered to have binding activity.

### 2.8. Cell Viability Assay

Briefly, human laryngeal epidermoid carcinoma (HEp-2) cells were cultured in RPMI 1640 containing 10% calf serum and were seeded (1 × 10^4^ cells) into the wells of a 96-well microplate and allowed to adhere for 24 h at 37 °C under 5% CO_2_. The cells were treated with suffruticosol A, suffruticosol B, suffruticosol C, paeonifloin, albiflorin, trans-ε-viniferin, gnetin H (Concentration for 1/2 MIC) for 10 min. Then, peony seed cake extract was washed away, and fresh culture medium was added prior to further incubate for 24 h. Cell viability was determined using an MTT Cell Proliferation and Cytotoxicity Assay Kit (C0009S, Beyotime, China) according to the manufacturer’s protocol. The cell survival rate is expressed as a percentage of the control value.

### 2.9. Statistic Methods

The significance of the data was analyzed according to unpaired Student’s two-sided t-test. * *p* < 0.05, ** *p* < 0.01, *** *p* < 0.001 and **** *p* < 0.0001. The samples were randomly allocated to experimental groups and processed for blind evaluation.

## 3. Results

### 3.1. MIC and MBC Value Results

**Figure 1:**
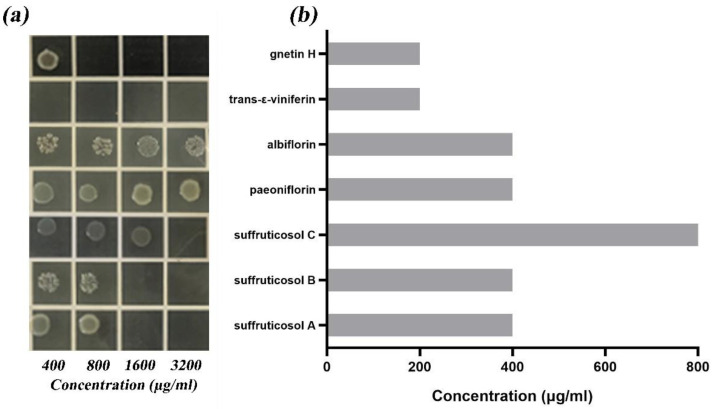
MIC and MBC values of each component of peony seed meal against *S. suis*. (**a**) MBC values (**b**) MIC values. In the figure, (**a**) MIC results and (**b**) MBC results are corresponding, which are suffruticosol A, suffruticosol B, suffruticosol C, albiflorin, paeonifloin, trans-ε-viniferin, gnetin H from bottom to top.

The results of MIC and MBC values are shown in Figure 1. The MIC/MBC values of the suffruticosol A, suffruticosol B, suffruticosol C, paeonifloin, albiflorin, trans-ε-viniferin, gnetin H were 400/1600, 400/1600, 800/3200, 400/>3200, 400/>3200, 200/200, and 200/400 μg/mL. Seven single components had no good inhibit or kill *S. suis*. 

### 3.2. Inhibition of Bacterial Biofilm

**Figure 2:**
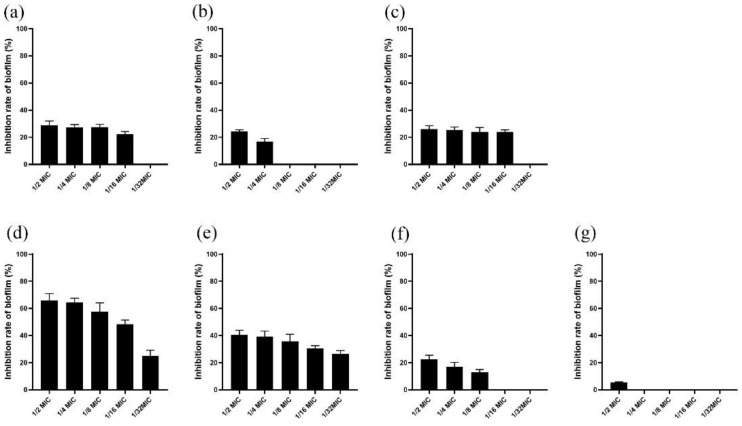
Biofilm formation by *S. suis* in the presence of sub-inhibitory concentrations of each component of peony seed meal. Biofilm was quantified by crystal violet staining following bacterial growth. From (**a**) to (**g**): (**a**): suffruticosol A; (**b**): suffruticosol B; (**c**): suffruticosol C; (**d**): paeonifloin; (**e**): albiflorin; (**f**): trans-ε-viniferin; (**g**): gnetin H. Data are shown as the mean ± SD of triplicate experiments.

The effect of sub-MIC concentration of peony seed cake extract on the formation of *S. suis* biofilm was studied. The results showed a concentration dependence, that is, with the decrease in the concentration of peony seed meal, the inhibitory ability of *S. suis* biofilm also decreased. As shown in Figure 2, the BF formation ability of *S. suis* was inhibited by the addition of a single component of peony seed cake extract at a concentration of ½ MIC compared with *S. suis* without drugs. Among them, the inhibition rates of biofilm of the suffruticosol A, suffruticosol B, suffruticosol C, paeonifloin, albiflorin, trans-ε-viniferin, gnetin H were 28.88%, 24.24%, 26.08%, 65.79%, 40.41%, 22.58%, 5.47%. The results showed that paeoniflorin had the most significant inhibitory effect on *S. suis* biofilm, and the inhibitory effect was concentration-dependent.

### 3.3. Effects on Biofilm Structure

**Figure 3:**
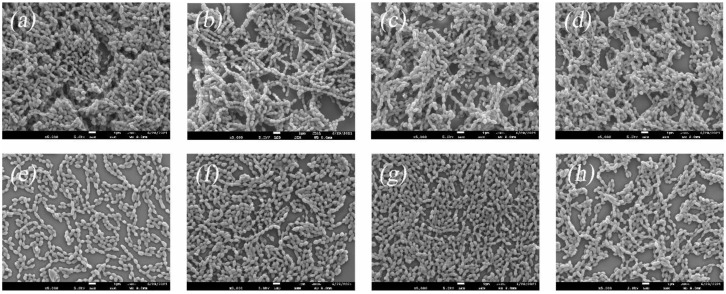
Scanning electron microscopy of *S. suis* biofilms; scale bars: 1 µm. From (**a**) to (**h**): (**a**): drug free control; (**b**): suffruticosol A; (**c**): suffruticosol B; (**d**): suffruticosol C; (**e**): paeonifloin; (**f**): albiflorin; (**g**): trans-ε-viniferin; (**h**): gnetin H.

The biofilm of *S. suis* was observed by scanning electron microscope. The results were shown below, as shown in Figure 3. The biofilm of the drug-free control group was rich, and the structure was complete, but the biofilm of each drug group showed different degrees of disintegration, among which the paeoniflorin group had the most significant degree of disintegration. The results showed that all the peony seed meal extracts could inhibit the formation of biofilm, and paeoniflorin significantly destroyed the biofilm structure of *S. suis*.

### 3.4. Determination of AI-2 Signaling Molecules

**Figure 4:**
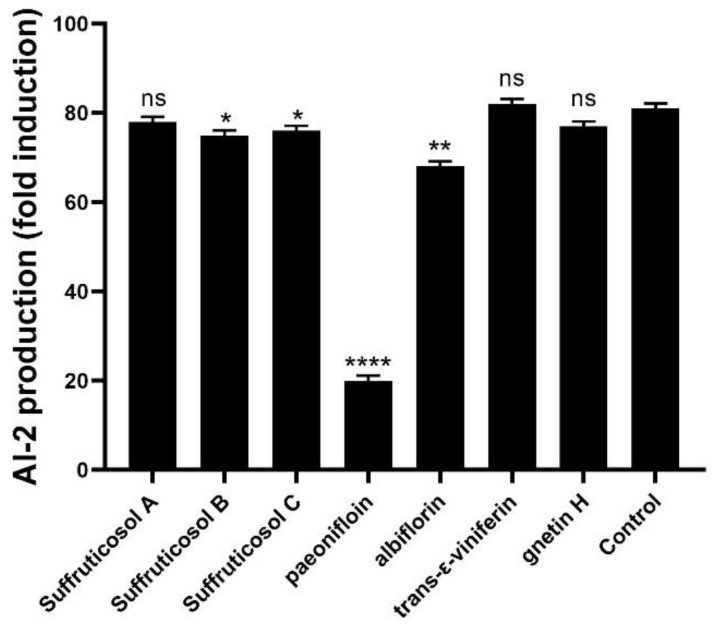
Suffruticosol A, suffruticosol B, suffruticosol C, paeonifloin, albiflorin, trans-ε-viniferin, gnetin H and control inhibits the secretion of AI-2 signaling molecule by *S. suis*. Data are shown as the mean ± SD. Statistical significance was assessed by unpaired Student’s two-sided *t*-test compared to the control group. * *p* < 0.05, ** *p* < 0.01, **** *p* < 0.0001. All experiments were performed in triplicate.

The effects of seven peony seed cake extracts on the production of AI-2 signaling molecules in *S. suis* were tested. The AI-2 production values of the suffruticosol A, suffruticosol B, suffruticosol C, paeonifloin, albiflorin, trans-ε-viniferin, gnetin H and control were 78, 75, 76, 20, 68, 82, 77, 81 (Figure 4). The results showed that paeoniflorin can significantly inhibit the production of AI-2 signaling molecules in *S. suis*.

### 3.5. Real-Time RT-PCR

**Figure 5:**
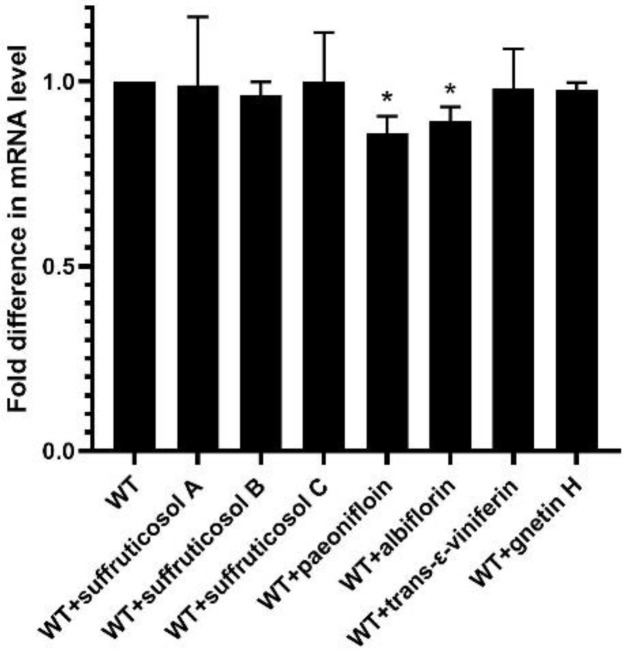
Relative expression of *luxS* genes. The figure shows that the gene expression level in the WT strain is 100%. Data from three independent assays are expressed as mean ± SD. Statistical significance was assessed by unpaired Student’s two-sided *t*-test compared to the control group. * *p* < 0.05.

Real-time PCR was used to compare the expression of *luxS* gene related to the LuxS/AI-2 system of *S. suis* after adding 1/2 MIC peony seed cake extract. The expression of *luxS* gene was significantly downregulated after the addition of paeoniflorin and albiflorin (Figure 5).

### 3.6. Molecular Docking Results

**Figure 6:**
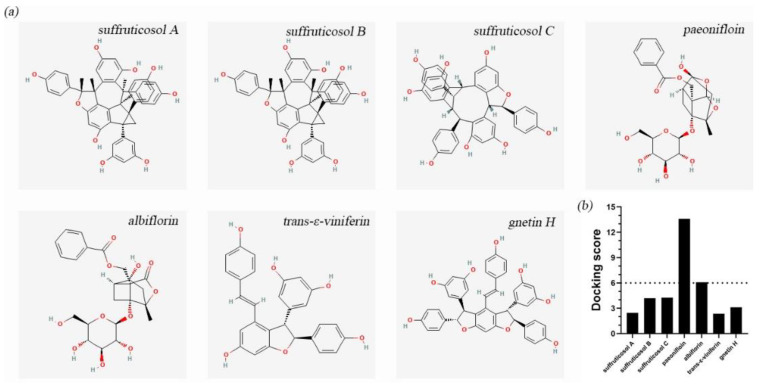
Interactions between each peony seed cake extract on LuxS. (**a**) 2D structure of each peony seed cake extract. (**b**) The binding fraction of each peony seed cake extract and LuxS protein, The dashed line indicates the lowest binding fraction.

In order to understand the interaction between each peony seed cake extract and LuxS, we performed a virtual docking experiment. As shown in Figure 6b, the binding scores of suffruticosol A, suffruticosol B, suffruticosol C, paeonifloin, albiflorin, trans-ε-viniferin, gnetin H and LuxS were, respectively, 2.45, 4.19, 4.25, 13.6, 6.07, 2.36, 3.12.

### 3.7. Cell Activity

**Figure 7:**
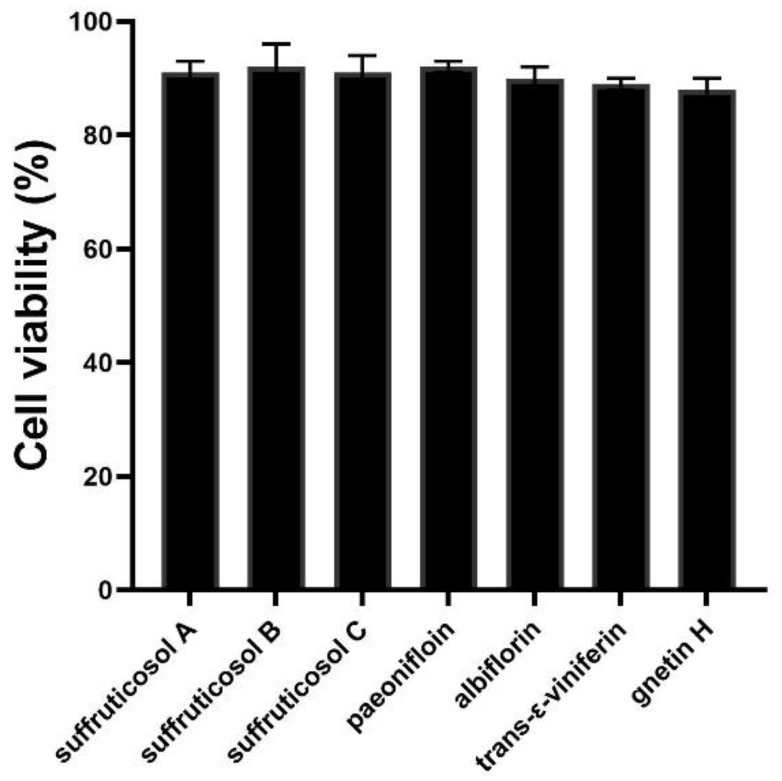
Influence of each peony seed cake extract on the viability of Hep-2 cells.

MTT assay was used to detect the effects of each peony seed cake extract on the survival rate of Hep-2 cells. As shown in Figure 7, there was no significant difference in the survival rate of Hep-2 cells under ½ MIC concentrations of suffruticosol A, suffruticosol B, suffruticosol C, paeonifloin, albiflorin, trans-ε-viniferin, gnetin H.

## 4. Discussion

*Streptococcus suis* is a Gram-positive facultative anaerobe that colonizes the upper respiratory tract, intestinal tract, and reproductive tract of pigs, causing acute or chronic infections and aggravating the disease [17]. Currently, antibiotics are the most effective tools for controlling *S. suis* infection. However, drug-resistant strains of *S. suis*, including those resistant to fluoroquinolones (FQs), have emerged as a result of the abuse and misuse of many antibiotics, such as tetracycline, sulfonamides, macrolides, β-lacamides, and fluoroquinolones [18,19]. The resistance and tolerance of *S. suis* are affected by many factors, including drug resistance gene mutations, metabolic level changes, horizontal gene transfer, bacterial outer vesicles, efflux pump, quorum sensing system regulation, and biofilm formation [20,21,22]. Studies have shown that quorum sensing can regulate a variety of drug resistance factors, biofilm, glucose metabolism level, bacterial outer vesicles, and efflux pumps, etc. [23,24].

Quorum-sensing system [5,25] and biofilm [26,27], as one of the main factors leading to bacterial resistance, promote the proliferation of drug-resistant bacteria and pose a serious threat to the pig industry. Therefore, there is an urgent need to find alternative ways to fight infections and reduce the emergence of resistant bacteria. Plants are the most extensive source of natural products, some of which have been found to have anti-quorum sensing systems and anti-biofilm properties. It has been found that essential oils (EOs) strongly inhibit biofilm formation and violin (QS) production in a concentration-dependent manner [28]. In addition, *Melaleuca bracteata* leaves EOs has potential bacteriostatic and QS inhibitor (QSI) effects on pathogens, which can prevent and control bacterial contamination [29]. At present, the research on plant inhibition of quorum sensing systems is extensive, but the research is usually limited to plant extract complexes, whose specific functional components are not clear.

In this study, the anti-Luxs /AI-2 system and anti-biofilm ability of seven main components of peony seed cake: suffruticosol A, suffruticosol B, suffruticosol C, paeonifloin, albiflorin, trans-ε-viniferin, gnetin H against *S. suis* were determined. The MIC and MBC results showed that the extract of peony seed cake did not have significant antibacterial and bactericidal effects. This is not the only development; the plant extract can be up to a milligram of bacteriostatic ability. Studies have found that the MIC value of Trollius altaicus C.A. Mey (TA) extract against l of *Streptococcus mutans* is 10 mg/mL [12]. However, surprisingly, further investigation revealed that paeoniflorin significantly reduced BF formation in *S. suis* at subminimum inhibitory concentration (1/2 MIC). MTT results showed that seven single components of peony seed meal had no obvious toxicity to Hep-2 cells at the concentration of 1/2 MIC, which proved its potential medicinal value. It has been found that Cinnamomum camphora leaf essential oil can inhibit BF by interfering with the bacterial QS system [30]. Medicinal herb Cassia alata has been reported to interfere with bacterial QS [31]. In addition, the subminimum inhibitory concentration (sub-MIC) of rhubarb water extract can inhibit BF of *S. suis* by inhibiting histidine kinase and histidine kinase two-component signal transduction system (TCSs) component proteins and response regulators [32]. Similar to other pathogens, *S. suis* exhibits virulence factors controlled by the QS system, such as BF formation, which complicates treatment. Therefore, attenuating the QS cycle involving LuxS/AI-2 in *S. suis* may be a promising alternative strategy to overcome *S. suis* infection. We determined the inhibitory effects of seven peony seed meal single components on the AI-2 signaling molecules of *S. suis*, and the results showed that paeoniflorin decreased the AI-2 activity in *S. suis* BF. In *S. suis*, the LuxS protease in the methyl cycle regulates AI-2 synthesis. Further measurement showed that paeoniflorin and albiflorin could inhibit the transcription level of *luxS* gene of *S. suis*, but not obviously. Molecular docking showed that paeoniflorin and LuxS protein had strong binding activity. The present results support our hypothesis that the major components of Paeoniflora can inhibit the synthesis of AI-2 signaling molecules by binding to LuxS protein to compete for effective active sites, thus weakening the quorum sensing system of LuxS/AI-2. However, it is not ruled out that the major components of Paeonia lactiflora can bind to AI-2 molecules and change the quorum sensing of AI-2 signaling molecules in bacteria. However, the specific influencing mechanism needs further experimental verification.

## 5. Conclusions

The inhibitory effects of seven single components of peony seed meal on *S. suis* BF and AI-2 signaling molecules were investigated in this study. The results showed that paeoniflorin had the most significant inhibitory ability on *S. suis* BF and AI-2 signaling molecules. These results provide a preliminary basis for further research on the regulation of *S. suis* by paeoniflorin through the LuxS/AI-2 QS system and provide new ideas for the prevention and control of *S. suis*.

## Data Availability

The original contributions presented in the study are included in the article; further inquiries can be directed to the corresponding authors.

## Supplementary Materials

The following supporting information can be downloaded at: https://www.mdpi.com/article/10.3390/ijerph192316024/s1, Table S1: Primers for qPCR used in this study.
