# Supplementary material for: Inhibitory Effect of Monoterpenoid Glycosides Extracts from Peony Seed Meal on Streptococcus suis LuxS/AI-2 Quorum Sensing System and Biofilm
Source: Int J Environ Res Public Health. 2022 Nov 30;19(23):16024. doi: 10.3390/ijerph192316024 (PMC9740070; doi:10.3390/ijerph192316024)
Supplement: Supplementary file 1 [file ijerph-19-16024-s001.zip › ijerph-2027685-supplementary.pdf]

Table S1. Primers for qPCR used in this study.

Name	Sequence (5′-3′)	Target gene
16s RNA-S	GTTGCGAACGGGTGAGTAA	*16sRNA*
16s RNA-A	TCTCAGGTCGGCTATGTATCG	
luxS-S	GGAAACCTGTCCGACACCC	*luxS*
luxS-A	TTCGCCTTATCCAACCCAA	
